# The effects of weight loss and improved metabolic health status on the risk of non-alcoholic fatty liver disease—results from a prospective cohort in China

**DOI:** 10.3389/fnut.2023.1239996

**Published:** 2023-11-28

**Authors:** Xin Huang, Wenbin Ouyang, Yang Hu, Bei Tang, Yongmei He, Hao Wu, Pingting Yang, Lu Yin, Qingqi Liu, Kui Chen, Jing Deng, Xiaohui Li, Ying Li

**Affiliations:** ^1^Department of Epidemiology, Hunan Normal University School of Medicine, Changsha, China; ^2^Department of Health Management, Aerospace Center Hospital, Beijing, China; ^3^Department of Health Management, The Third Xiangya Hospital, Central South University, Changsha, China; ^4^Medical Research and Biometrics Center, National Center for Cardiovascular Diseases, Chinese Academy of Medical Sciences and Peking Union Medical College, Beijing, China; ^5^Department of Biostatistics, Bioinformatics and Biomathematics, Georgetown University, Washington, DC, United States; ^6^Department of Epidemiology, Xiangya School of Public Health, Central South University, Changsha, China; ^7^Department of Pharmacology, Xiangya School of Pharmaceutical Science, Central South University, Changsha, Hunan, China

**Keywords:** obesity, metabolic health, NAFLD, cohort study, loss weight

## Abstract

**Background:**

The impact of weight loss and/or improved metabolic status on the risk of non-alcoholic fatty liver disease (NAFLD) has yet to be determined.

**Methods:**

A total of 35,322 participants without NAFLD were followed. NAFLD risk was compared between consistently metabolically healthy non-obese (MHNO) and non-MHNO who lost weight to become non-obese and/or improved their metabolic health, using Cox proportional hazards and logistic regression models.

**Results:**

Following 148,186 person-years, 8,409 participants had onset NAFLD, with an incidence rate of 56.75 (95% CI: 55.57, 57.94) per 1,000 person-years. Metabolically healthy obese (MHO), metabolically unhealthy obese (MUO), and metabolically unhealthy non-obese (MUNO) at baseline were associated with increased NAFLD risk, with hazard ratios of 4.48 (95%CI:4.24, 4.73), 8.85 (95%CI:7.95, 9.84), and 10.70 (95%CI:9.73, 11.78). Weight loss and/or metabolic status improvements could significantly reduce NAFLD risk by 79.46 to 41.46%. Specifically, after weight loss from MHO to MHNO, the reduction in NAFLD risk [OR decreased from 12.01 (95%CI:9.40, 15.35) to 4.14 (95%CI:3.08, 5.57)] was greater than that of the MUNO subgroup whose metabolic status improved to MHNO [OR decreased from 5.53 (95%CI:5.15, 5.94) to 2.71 (95%CI:2.50, 3.93)]. In the MUO subgroup, the group with the greatest risk reduction of NAFLD was the weight and metabolic state both improvement group [MUO to MHNO, OR decreased from 22.74 (95%CI:17.61, 29.37) to 4.67 (95%CI:3.05, 7.16)], followed by the weight loss only group [MUO to MUNO, OR decreased to 6.83 (95%CI:4.87, 9.57)], and finally the group with the least and insignificant risk reduction was the metabolic state improvement group [MUO to MHO, OR decreased to 13.38 (95%CI:9.17,19.53)]. NAFLD risk was negatively correlated with the duration of improvement (*p* < 0.001).

**Conclusion:**

Individuals with non-MHNO were more likely to develop NAFLD than those with consistent MHNO, but metabolic improvements and weight loss can alleviate the risk. Their NAFLD risk was negatively correlated with improvement duration. However, it remained higher than in individuals with consistent MHNO at an average follow-up of 4.2 years.

## Introduction

Non-alcoholic fatty liver disease (NAFLD) is the most common chronic liver disease and a risk factor for cardiovascular disease and death worldwide (1–4). It is estimated that the global prevalence of NAFLD was approximately 25.2% (5). Obesity, insulin resistance, and sub-clinical inflammation are considered the major determinants of NAFLD. The presence of obesity, metabolic abnormalities, or inflammatory abnormalities alone or in combination can increase the risk of NAFLD (6–9). Additionally, obesity is frequently accompanied by metabolic syndrome (MetS), which refers to abdominal obesity, elevated blood pressure, fasting glucose and triglycerides, and low levels of high-density lipoprotein cholesterol (HDL-c). MetS and NAFLD are closely linked, and both contribute to each other. NAFLD is now recognized as a cause and consequence of MetS, especially diabetes. The relationship between NAFLD and diabetes is more complex than previously thought. First, NAFLD can help assess the risk of complications in diabetes based on new findings. Second, NAFLD should be included in disease management programs for diabetic patients. Third, NAFLD should be considered when personalizing diabetes treatment, as it shares its underlying causes with certain forms of prediabetes and diabetes (8, 10–13).

Depending on the presence or absence of MetS and obesity, the population could be classified into the following four phenotypes: metabolically healthy non-obese (MHNO); metabolically healthy obese (MHO); metabolically unhealthy non-obese (MUNO); and metabolically unhealthy obese (MUHO). Different phenotypes present different NAFLD risks (14–16), liver fibrosis progression (17), and mortality due to all causes (18). In addition, recent studies suggest that NAFLD risk may be affected by changes in phenotype. Chang et al. (15) followed 77,425 metabolic health participants for 4.5 years and found that increasing BMI was independently associated with an increased risk of NAFLD. Cho et al. (19) followed 14,779 metabolically healthy overweight or obese (BMI ≥ 23 kg/m^2^) participants for 5.2 years and found that clinically relevant weight loss of >5% was associated with a lowered risk of NAFLD. Those prior studies, however, focused mainly on metabolically normal populations. It remains unclear whether subjects with unhealthy metabolic conditions, such as the MUNO or MUO phenotype, might benefit from weight loss and improved metabolic status, thereby reducing their risk of NAFLD.

Conducting studies on the association between metabolic status improvement, weight loss, and NAFLD risk among metabolically abnormal populations will help us better understand clinical expectations for lifestyle and weight interventions. According to our previous research (20), more than one-third of Chinese had metabolic abnormal or obesity problems in 2012, including 3.47% of MHO, 21.93% of MUNO, and 5.36% of MUO, which is a particularly prominent public health issue. We, therefore, have followed Chinese subjects without NAFLD since 2012 to investigate whether weight loss and/or improved metabolic status could reduce the risk of NAFLD in individuals with different obesity and metabolic health statuses.

## Patients and methods

### Study population

Participants in the cohort study were enrolled in the health management centers of Aerospace Center Hospital (in Beijing) and the Third Xiangya Hospital (in Hunan). It was common for people from local cities to visit these two hospitals, as well as people from nearby suburban counties. Those who underwent annual health check-ups in those two centers between January 2012 and December 2018 were eligible for selection. Aiming at exploring the association between transition in metabolic and obese phenotypes and NAFLD risk, subjects without data for exposure or outcome assessment were, therefore, excluded. The specific inclusion criteria were: (1) aged > 20 years, not pregnant at baseline and consent to participate; (2) having at least 2 follow-ups before December 2020; (3) with liver ultrasound results both at baseline and at follow-ups; (4) having enough data for metabolic and obesity status assessment at baseline and during follow-up periods; (5) free of NAFLD, hepatic fibrosis or cyst, or hepatic viral infection, and not using medication for the above listed diseases at baseline enrollment. To avoid selection bias resulting from missed diagnoses at baseline, those diagnosed with NAFLD, hepatic cysts, or fibrosis in the first 12 months following enrollment were further excluded. Approximately 80% of the participants in Hunan were from urban areas, while 95% of the participants in Beijing came from urban areas. All participants had signed informed consent forms, and the Third Xiangya Hospital Ethics Committee approved the study.

### Measurement and definition

Typical annual health check-ups in those two health management centers consisted of a questionnaire, a series of physical examinations, and laboratory tests. Participants were asked to complete questionnaires detailing their age, gender, smoking history, alcohol consumption, current medications, and previous medical diagnoses. Physicians would confirm those details during the physical examination. Fasting venous blood samples were collected and analyzed at the clinical laboratory of the corresponding hospital for health check-ups. Methods used for physical and laboratory examinations were detailed in the online appendix, as was the definition of chronic diseases. Subjects with weight or metabolic health problems will receive general recommendations in their health check-up report, including weight control, physical activity enhancement, and nutrition clinic visits. In the nutrition clinic of our health management centers, experienced experts will offer personalized dietary structure adjustments and physical exercise recommendations to help control weight and improve metabolic health. Those suspected of having certain kinds of diseases might be referred to outpatient clinics for further diagnosis or treatment. Most people would have an annual physical examination every 8–16 months. It is possible for some individuals to have their examination earlier or later than that time interval due to special problems that require re-examinations or collective payments from their employers.

The primary outcome of our study was the onset of NAFLD during the follow-up period. A NAFLD diagnosis was established when transabdominal ultrasound showed signs of hepatic steatosis without secondary causes of liver fat accumulation, such as excessive alcohol consumption, long-term steatogenic medication use, or monogenic hereditary disorders. The sign of steatosis refers to the increased echogenicity of the liver parenchyma compared to the cortex of the right kidney. Excessive alcohol consumption was defined as consuming more than 25 grams of ethanol per day for men and more than 15 grams per day for women. All the participants in this study were followed up from the baseline enrollment index data until NAFLD diagnosis, withdrawal from the study, or the end of 2020.

The body mass index (BMI) range in our population was 15 to 40. According to the standard weight criteria for adults in China, a BMI ≥ 28 kg/m^2^ was defined as obesity. The MetS was defined as the presence of two or more of the following four conditions: (1) elevated triglyceridaemia (TG), TG ≥ 1.70 mmol/L or usage of lipid-lowering drugs; (2) low HDL-c, HDL-c < 1.04 mmol/L in male subjects or < 1.29 mmol/L in female subjects; (3) elevated blood pressure, systolic blood pressure (SBP) ≥ 130 mmHg, or diastolic blood pressure (DBP) ≥ 85 mmHg or usage of antihypertensive drugs; and (4) elevated fasting serum glucose (FSG), FSG ≥ 5.6 mmol/L, or usage of medications for diabetes. According to obesity and metabolic status, we divided participants into four phenotypes: MHNO, MUNO, MHO, and MUO. The phenotype classification was measured under the same definition at baseline and at all follow-up visits. During the follow-up period, one’s phenotype may not remain constant and could vary several times. Accordingly, four types of variation patterns were identified: “throughout” was defined as exhibiting no phenotype variation during the follow-up period; “ever” was defined as a change in phenotype during the follow-up period; “intermittent” was defined as the change in phenotype at a specific follow-up and returning to the original condition; whereas “consistent” was defined as the continued change in phenotype after a specific follow-up. The purpose of our study was to determine whether weight loss or improved metabolism could reduce the risk of NAFLD in the non-MHNO population to the same level as in the MHNO population. We, therefore, further excluded 6,044 participants who were MHNO at baseline and deteriorated to any other phenotype during follow-up, which yielded a final sample size of 35,322.

The need for medication for MetS was defined as the presence of one or more of the following conditions: TG ≥ 5.60 mmol/L or usage of lipid-lowering drugs, SBP ≥ 140 mmHg, or DBP ≥ 90 mmHg or usage of antihypertensive drugs, FSG ≥ 7.1 mmol/L or usage of medications for diabetes.

### Statistical analysis

The distributions of baseline characteristics were compared between different obesity and MetS phenotype groups. Cox proportional hazard models were used to estimate the associations between obesity and metabolic status at baseline and the risk of NAFLD and MHNO throughout the group as a reference. Potential confounding variables included age, sex, smoking, alcohol usage, and location of enrollment.

**Figure 1:**
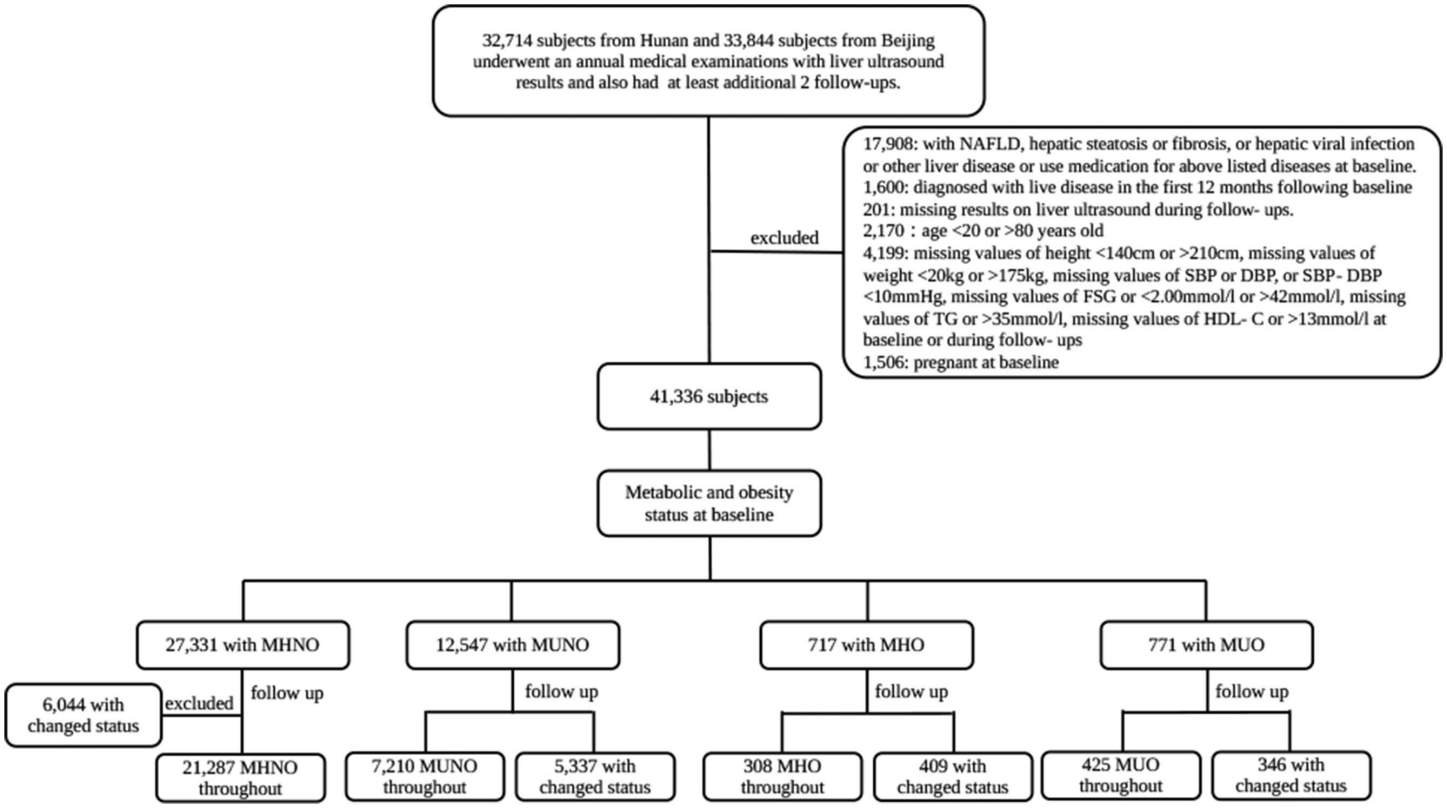
Enrolment flowchart. NAFLD, non-alcoholic fatty liver disease; MHNO, metabolic healthy non-obese; MUNO, metabolic unhealthy non-obese; MHO, metabolic healthy obese; MUO, metabolic unhealthy obese; BMI, body mass index; SBP, systolic blood pressure; DBP, diastolic blood pressure; LDL-C, low-density lipoprotein cholesterol; HDL-C, high-density lipoprotein cholesterol; TG, triglyceride; FSG, fasting serum glucose.

The exposures of interest in this study were improvements in obesity and metabolic phenotypes. In summary, the exposure categories included MUNO changes in MHNO, MHO changes in MHNO, MUO changes in MHO, MUO changes in MUNO, and MUO changes in MHNO. Meanwhile, MHNO throughout the group was considered non-exposed. The duration of improvement was calculated by adding half the interval between the time the improved phenotype was discovered and the time of the previous follow-up that was conducted, plus half the interval between the time when the improved phenotype was discovered and the time when the subsequent different phenotype appeared. For the “consistent” category, the length of exposure was calculated by adding half the time interval between the time when the improved phenotype was found and the time of the previous follow-up when no improvement occurred, plus the time between the time when the improved phenotype was found and the end of the subsequent follow-up period. For “intermittent” categories where improvements are observed at multiple intervals, cumulative exposure is calculated by adding the duration of each intermittent improvement. The improvement intervals are calculated by adding half the time between when a phenotype improved and the time when no improvement occurred in the previous follow-up, plus half the time between when the phenotype improved and when it changed. Logistic regression models were used to estimate the association between the risk of NAFLD and the exposure category, as well as the dose–response relationship. Sensitivity analysis was conducted by assessing the modification effect by age, sex, smoking, alcohol usage, location of enrollment, and need for medication for MetS and by using restricted cubic spline (RCS) logistic models to test the association between exposure duration and risk of NAFLD. Since the duration of the independent variable exposure in this model was measurement data, we used the RCS model to calculate the estimated dose–response relationship, in which the lowest dose reference group was those with no change in the phenotype from baseline to follow-up. A two-sided *p*-value of <0.05 was considered to be statistically significant. SAS version 9.4 (SAS Institute Inc.) was used for analyses (Figure 1).

## Results

Overall, we followed 35,322 participants with an average age of 41.38 years old for 12 to 96 months. Demographic and medical factors differed significantly between the obesity and metabolic phenotype groups (*p* < 0.001), except for the location of enrollment (*p* = 0.873; Table 1).

**Table 1:** Characteristics of enrolled participants by obesity and metabolic status at baseline (*n* = 35,322).

Characterization	MHNO (*n* = 21,287)	MUNO (*n* = 12,547)	MHO (*n* = 717)	MUO (*n* = 771)	*P*
	Mean (SD)/*N* (%)	Mean (SD)/*N* (%)	Mean (SD)/*N* (%)	Mean (SD)/*N* (%)	
Age	36.78(11.22)	49.08(14.83)	39.10(12.07)	45.65(14.67)	<0.001
Female	13,993(65.73)	4,022(32.06)	284(39.61)	162(21.01)	<0.001
Enrolled from Beijing	10,627(49.92)	6,268(49.96)	351(48.95)	394(51.10)	0.873
Smoking	4,317(20.28)	4,074(32.47)	228(31.80)	344(44.62)	<0.001
Alcohol usage	6,197(29.11)	4,820(38.42)	298(41.56)	398(51.62)	<0.001
Hypertension	499(2.34)	3,938(31.39)	83(11.58)	318(41.25)	<0.001
Diabetes mellitus	39(0.18)	943(7.52)	4(0.56)	80(10.38)	<0.001
Dyslipidemia	1,231(5.78)	5,926(47.23)	121(16.88)	446(57.85)	<0.001
BMI, Kg/m^2^	21.38(2.23)	23.62(2.20)	28.82(1.55)	29.22(1.54)	<0.001
SBP, mmHg	112.46(11.69)	130.91(15.55)	121.45(13.31)	134.72(15.41)	<0.001
DBP, mmHg	68.98(8.49)	79.61(10.70)	74.46(9.38)	82.83(11.24)	<0.001
Total cholesterol, mmol/L	4.61(0.82)	5.09(1.00)	4.84(0.87)	5.16(1.22)	<0.001
LDL-c, mmol/L	2.41(0.72)	2.72(0.87)	2.77(0.77)	2.77(0.91)	<0.001
HDL-c, mmol/L	1.76(0.38)	1.40(0.38)	1.46(0.32)	1.27(0.32)	<0.001
Triglycerides, mmol/L	0.95(0.43)	2.15(1.62)	1.31(0.67)	2.53(2.12)	<0.001
FSG, mmol/L	4.95(0.47)	5.72(1.33)	5.12(0.66)	5.89(1.72)	<0.001
≥1 component of the MetS requires medication	549(2.58)	4,697(37.44)	89(12.41)	373(48.38)	<0.001

MHNO, metabolic healthy non-obese; MUNO, metabolic unhealthy non-obese; MHO, metabolic healthy obese; MUO, metabolic unhealthy obese; BMI, body mass index; SBP, systolic blood pressure; DBP, diastolic blood pressure; LDL-c, low-density lipoprotein cholesterol; HDL-c, high-density lipoprotein cholesterol; FSG, fasting serum glucose; MetS, metabolic syndrome; SD, standard deviation.

**Figure 2:**
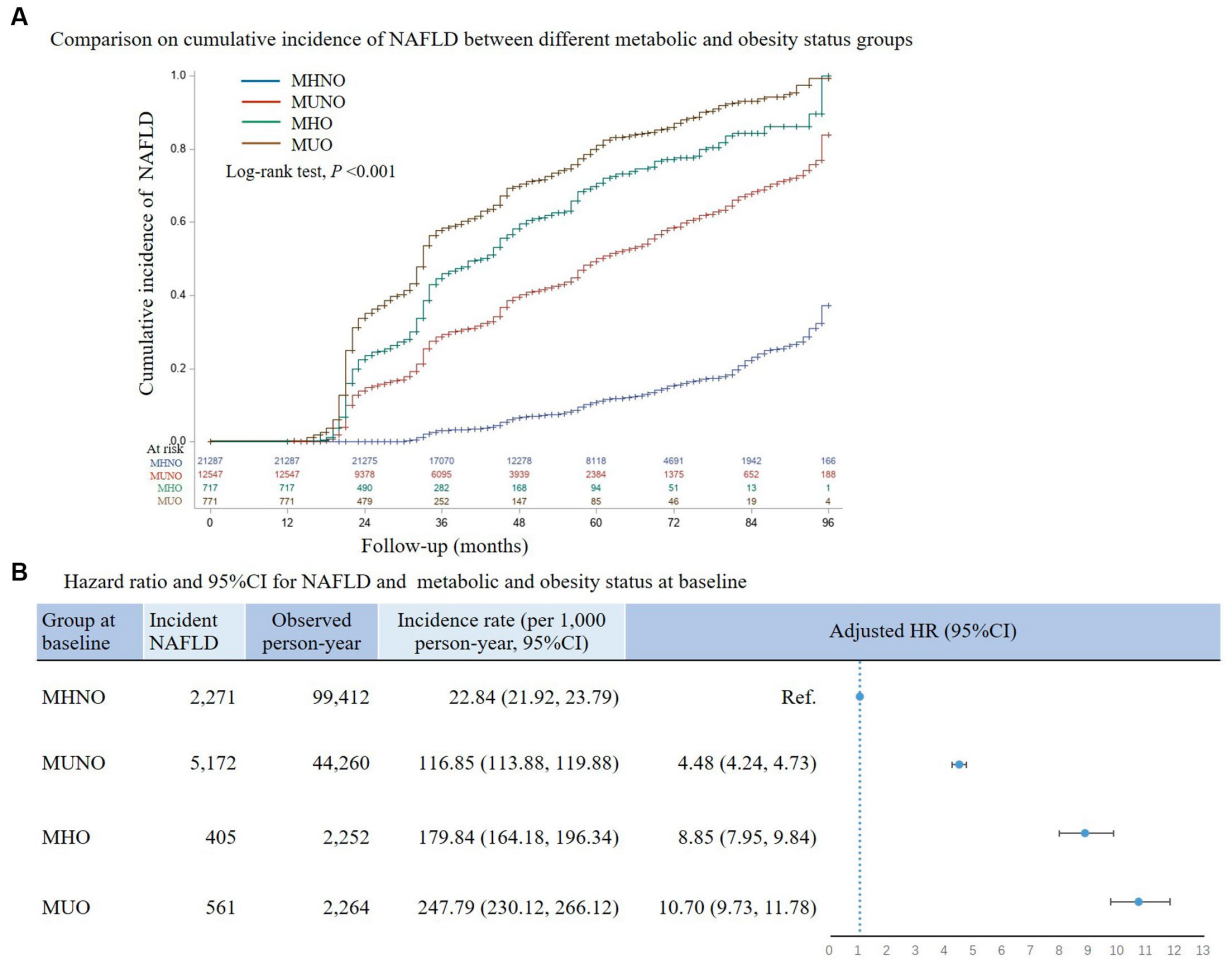
Association between risk of NAFLD and metabolic and obesity status at baseline. In panel **(A)** comparison between groups was based on Kaplan–Meier method; in panel **(B)** hazard ratio estimation was based on the cox proportion hazards model and adjustment variables included sex, age, alcohol usage, smoking, and location of enrollment. NAFLD, non-alcoholic fatty liver disease; MHNO, metabolic healthy non-obese; MUNO, metabolic unhealthy non-obese; MHO, metabolic healthy obese; MUO, metabolic unhealthy obese; HR, hazard ratio.

During 148,186 person-years of follow-up, 8,409 participants had onset NAFLD, with an incidence rate of 56.75 (95%CI:55.57, 57.94) per 1,000 person-years. The median follow-up duration was 47 months for the overall subjects, 36 months for those with the onset of NAFLD, and 48 months for those without. Significantly different NAFLD risks were found between different baseline phenotype groups (*p* < 0.001; Figure 2A). In comparison with the MHNO at baseline group, the hazard ratios for MUNO at baseline, MHO at baseline, and MUO at baseline were still significantly higher after adjustment for confounding, respectively, at 4.48 (95%CI:4.24, 4.73), 8.85 (95%CI:7.95, 9.84), and 10.70 (95%CI:9.73, 11.78; Figure 2B).

The results of logistics models are listed in Tables 2, 3. For participants with MUNO at baseline, the risk of NAFLD decreased significantly for those who ever improved to the MHNO phenotype [OR = 5.53 (95%CI:5.15, 5.94) decreased to 2.71 (95%CI:2.50, 2.93)], and the reduction was significantly higher in the group that improved consistently to the MHNO phenotype compared to the group that improved intermittently (*p* < 0.05; Table 2). For participants with MUO at baseline, the improvement of metabolic health to the MHO phenotype could also reduce the risk of NAFLD, and the risk of consistently changing to the MHO group was statistically different from the risk of MUO throughout the group [OR = 3.85 (95%CI:1.19, 12.50) vs. 22.74 (95%CI:17.61, 29.37)]. The improvement in metabolic health among MUNO at baseline or MUO at baseline could not completely reduce the risk of NAFLD to the level of those with MHNO at baseline (*p* < 0.05; Tables 2, 3). However, the duration of improvement in metabolic health in the MUNO and MUO groups both showed a significantly linear association with lower odds of NAFLD (*P* for trend < 0.001; Table 3).

**Table 2:** Association between NAFLD and improvement in metabolic health and weight loss during follow-up.

Exposure group	NAFLD, *n* (%)		Crude OR (95%CI)	Adjusted OR (95%CI)^┼^
	Control	Case		
MHNO throughout (*n* = 21,287)	19,016(89.33)	2,271(10.67)	1.00	1.00
**MUNO at baseline (*n* = 12,547)**				
MUNO throughout (*n* = 7,210)	3,805(52.77)	3,405(47.23)	7.49(7.03,7.98)	5.53(5.15,5.94)
Ever MUNO to MHNO (*n* = 5,032)	3,514(69.83)	1,518(30.17)	3.62(3.36,3.90)	2.71(2.50,2.93)
Intermittent MUNO to MHNO (*n* = 3,924)	2,668(67.99)	1,256(32.01)	3.94(3.64,4.27)	2.95(2.71,3.21)
Consistent MUNO to MHNO (*n* = 1,108)	846(76.35)	262(23.65)	2.59(2.24,3.00)	1.97(1.70,2.28)
**MHO at baseline (*n* = 717)**				
MHO throughout (*n* = 308)	116(37.66)	192(62.34)	13.86(10.96,17.52)	12.01(9.40,15.35)
Ever MHO to MHNO (*n* = 214)	137(64.02)	77(35.98)	4.71(3.55,6.24)	4.14(3.08,5.57)
Intermittent MHO to MHNO (*n* = 161)	104(64.60)	57(35.40)	4.59(3.31,6.36)	3.79(2.69,5.33)
Consistent MHO to MHNO (*n* = 53)	33(62.26)	20(37.74)	5.08(2.91,8.86)	5.48(3.04,9.87)
**MUO at baseline (*n* = 771)**				
MUO throughout (*n* = 425)	79(18.59)	346(81.41)	36.67(28.61,47.01)	22.74(17.61,29.37)
Ever MUO to MHNO (*n* = 94)	51(54.26)	43(45.74)	7.06(4.69,10.62)	4.67(3.05,7.16)
Intermittent MUO to MHNO (*n* = 82)	41(50.00)	41(50.00)	8.37(5.42,12.94)	5.48(3.48,8.63)
Consistent MUO to MHNO (*n* = 12)	10(83.33)	2(16.67)	1.68(0.37,7.65)	1.16(0.24,5.55)
Ever MUO to MUNO (*n* = 154)	62(40.26)	92(59.74)	12.43(8.98,17.20)	6.83(4.87,9.57)
Intermittent MUO to MUNO (*n* = 118)	47(39.83)	71(60.17)	12.65(8.73,18.33)	6.77(4.61,9.94)
Consistent MUO to MUNO (*n* = 36)	15(41.67)	21(58.33)	11.72(6.04,22.77)	7.02(3.52,14.02)
Ever MUO to MHO (*n* = 140)	42(30.00)	98(70.00)	19.54(13.57,28.11)	13.38(9.17,19.53)
Intermittent MUO to MHO (*n* = 127)	34(26.77)	93(73.23)	22.90(15.43,34.01)	15.48(10.28,23.30)
Consistent MUO to MHO (*n* = 13)	8(61.54)	5(38.46)	5.23(1.71,16.01)	3.85(1.19,12.50)

^┼^Adjustment variables included sex, age, alcohol usage, smoking, location of enrollment, and LDL cholesterol. NAFLD, non-alcoholic fatty liver disease; MHNO, metabolic healthy non-obese; MUNO, metabolic unhealthy non-obese; MHO, metabolic healthy obese; MUO, metabolic unhealthy obese; OR, odds ratio; CI, confidence interval.

**Table 3:** Association between NAFLD and duration of improvement in weight and metabolic health during follow-up.

Exposure duration	NAFLD, *n* (%)		Crude OR (95%CI)	*P* for trend	Adjusted OR (95%CI)^┼^	*P* for trend
	Cases	Control				
MHNO throughout (*n* = 21,287)	2,271(10.67)	19,016(89.33)	1.00		1.00	
**Changed from MUNO to MHNO**						
0 months (*n* = 7,210)	3,805(52.77)	3,405(47.23)	7.49(7.03,7.98)	<0.001	5.53(5.15,5.94)	<0.001
1–12 months (*n* = 1,543)	495(32.08)	1,048(67.92)	3.96(3.52,4.44)		2.71(2.40,3.06)	
13–24 months (*n* = 1,793)	574(32.01)	1,219(67.99)	3.94(3.54,4.39)		2.62(2.33,2.94)	
25-months (*n* = 1,696)	449(26.47)	1,247(73.53)	3.02(2.68,3.39)		2.12(1.88,2.39)	
**Changed from MHO to MHNO**						
0 months (*n* = 308)	116(37.66)	192(62.34)	13.86(10.96,17.52)	<0.001	12.01(9.40,15.35)	<0.001
1–12 months (*n* = 71)	26(36.62)	45(63.38)	4.84(2.98,7.86)		4.33(2.61,7.19)	
13–24 months (*n* = 76)	27(35.53)	49(64.47)	4.61(2.88,7.40)		3.59(2.19,5.89)	
25-months (*n* = 67)	24(35.82)	43(64.18)	4.67(2.83,7.72)		4.67(2.75,7.93)	
**Changed from MUO to MHNO**						
0 months (*n* = 425)	79(18.59)	346(81.41)	36.67(28.61,47.01)	<0.001	22.74(17.61,29.37)	<0.001
≤12 months (*n* = 39)	17(43.59)	22(56.41)	6.47(3.43,12.20)		4.31(2.22,8.35)	
13-months (n = 55)	26(47.27)	29(52.73)	7.51(4.41,12.77)		4.89(2.80,8.54)	
**Changed from MUO to MUNO**						
0 months (*n* = 425)	79(18.59)	346(81.41)	36.67(28.61,47.01)	<0.001	22.74(17.61,29.37)	<0.001
≤12 months (*n* = 60)	36(60.00)	24(40.00)	12.56(7.48,21.09)		6.27(3.68,10.68)	
13–24 months (*n* = 62)	38(61.29)	24(38.71)	13.26(7.94,22.14)		8.45(4.94,14.46)	
25-months (*n* = 32)	18(56.25)	14(43.75)	10.77(5.35,21.68)		5.06(2.47,10.38)	
**Changed from MUO to MHO**						
0 months (*n* = 425)	79(18.59)	346(81.41)	36.67(28.61,47.01)	<0.001	22.74(17.61,29.37)	<0.001
≤12 months (*n* = 64)	51(79.69)	13(20.31)	32.85(17.84,60.49)		24.20(12.87,45.53)	
13–24 months (*n* = 52)	35(67.31)	17(32.69)	17.24(9.64,30.82)		11.01(6.03,20.11)	
25 months (*n* = 24)	12(50.00)	12(50.00)	8.37(3.76,18.66)		5.33(2.31,12.31)	

^┼^Adjustment variables included sex, age, alcohol usage, smoking, location of enrollment location, and LDL cholesterol. NAFLD, non-alcoholic fatty liver disease; MHNO, metabolic healthy non-obese; MUNO, metabolic unhealthy non-obese; MHO, metabolic healthy obese; MUO, metabolic unhealthy obese; OR, odds ratio; CI, confidence interval.

Population with improved obesity status, including changing from MHO to MHNO group and changing from MUO to MUNO group, also exhibited a reduced risk of NAFLD compared with the risk in the unchanged phenotype group (*p* < 0.05; Table 2), as well as a negative dose–response relationship between the duration of improvement and risk of NAFLD (*P* for trend < 0.001; Table 3).

Participants changing from MUO to MHNO involved simultaneous improvements in obesity and metabolic status, and a significantly lower NAFLD risk than the MUO throughout the group was observed (*p* < 0.05). The risk of NAFLD in the consistent improvement of the MUO group was not statistically different from that in the MHNO group, with OR = 1.16 (95%CI:0.24, 5.55). Less than five subjects had a cumulative improvement in MHNO greater than 24 months. Therefore, we only estimated the odds ratios for those with <12 months of cumulative improvement and ≥12 months, and no statistical difference was found between those two subgroups.

### Sensitivity analyses

**Figure 3:**
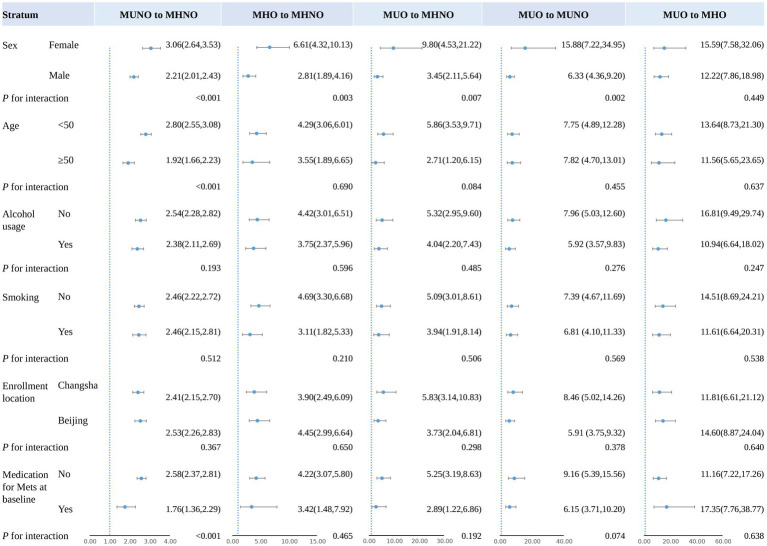
Sensitivity analysis for the association between NAFLD and improvement in weight and metabolic health during follow-up. All the adjusted odd ratios in the figure were calculated using MHNO throughout the group as the reference group. The adjustment variables included sex, age, alcohol usage, smoking, location of enrollment, and medication for metabolic syndrome components at baseline. When stratified by one of these factors, the stratification variable would not be included in the model. NAFLD, non-alcoholic fatty liver disease; MHNO, metabolic healthy non-obese; MUNO, metabolic unhealthy non-obese; MHO, metabolic healthy obese; MUO, metabolic unhealthy obese; MetS, metabolic syndromes; OR, odds ratio; CI, confidence interval.

The RCS logistic model observed similarly significant associations between the duration of improvement in obesity and metabolic status and reduced risk of NAFLD (*p* < 0.001; Supplementary Figure S1). Except for changes from MUO to MUNO or MHO, those dose–response relationships were non-linear (*p* < 0.05). In male subjects, improvements in obesity or metabolic status resulted in lower risks of NAFLD compared to female subjects (*p* < 0.01), with the exception of changing from the MUO to the MHO group (*p* = 0.449). Among participants who changed from MUNO to MHNO, the risk of NAFLD was lower among those over 50 than among those <50 [OR = 1.92 (95%CI:1.66, 2.23) vs. 2.80 (95%CI:2.55, 3.08)]. We also find heterogeneity in the risk of NAFLD due to the need for MetS medication at baseline. Participants with metabolic improvement who changed from MUNO to MHNO and required medication for MetS at baseline had a lower risk of NAFLD than those who did not need medication [OR = 1.76 (95%CI:1.36, 2.29) vs. 2.58 (95%CI:2.37, 2.81, *p* < 0.001); Figure 3].

## Discussion

Our study compared NAFLD risk in populations with different metabolic health and obesity statuses. It also examined whether NAFLD risk changed with improvements in metabolic and obesity status. As expected, we found that individuals with MHO, MUNO, and MUO have a higher incidence of NAFLD than MHNO. In addition, we demonstrated for the first time that clinically intermittent or consistent improvements in BMI and/or metabolic status were significantly associated with a reduced risk of NAFLD.

In this large cohort study, metabolic unhealthy and obesity status at baseline were found to be associated with a higher risk of NAFLD. In a retrospective study, Wu et al. (21) reported that the prevalence of NAFLD was 7.14%, 27.92%, 34.80%, and 61.02% in MHNO, MUNO, MHO, and MUO in elderly Chinese adults, respectively. To our knowledge, this is the first cohort study reporting the incidence of NAFLD among Chinese adults. MUNO, MHO, and MUO groups had approximately 4-fold, 8-fold, and 10-fold higher risks of developing NAFLD than MHNO. Our results indicated that obesity has a greater impact on NAFLD incidence than metabolic abnormalities. Moreover, obesity combined with abnormal metabolism has the greatest impact on NAFLD incidence.

Emerging epidemiological evidence indicates that MHO increases NAFLD risk significantly. For example, in Vusirikala’s study, MHO individuals had an approximately 7-fold higher risk of NAFLD than the MHNO population. This was a retrospective, population-based longitudinal cohort study conducted using over 4 million primary care patient records in the UK (16), and the results were similar to those of our own study. Moreover, BMI has shown a strong linear relationship with NAFLD incidence (15). These studies suggest obesity could significantly increase NAFLD risk, regardless of metabolic status. In metabolically healthy individuals, obesity increases the risk of fatty liver disease through the following mechanisms: (1) increased free fatty acids (FFAs), proinflammatory cytokines, ceramide, and dysregulated adipokine secretion from adipose tissue may contribute to hepatic lipid storage (22); and (2) MHO showed reduced cardiorespiratory fitness (CRF) compared to MHNO. And CRF was inversely associated with liver fat content and insulin resistance (23).

In the course of a person’s life, the MHO phenotype might change and evolve. The proportion of metabolically healthy individuals who deteriorate varies depending on their ethnicity, gender, age, and follow-up period. Approximately 23%–74% of metabolically healthy individuals develop metabolic abnormalities at some point in their lives (24–27). However, few studies focus on the impact of MHO improvement on the risk of NAFLD. In our study, after an average of 4.2 years of follow-up, approximately 30% of individuals experienced improvement from MHO to MHNO, which provides novel evidence for an improving trend in obesity among the Chinese health check-up population. It is worth noting that individuals in this study may have attended nutrition clinics or been referred to outpatient clinics after their annual health check-up. In these clinics, patients could receive personalized dietary and exercise advice or medicine prescriptions. Consequently, the rate of obesity reduction and metabolic improvement in the current study may not be generalizable to the general population. Previous studies reported that the transition from normal weight to obesity was associated with a 2-fold increase in NAFLD risk among metabolically health individuals (15). Since there are many obese individuals who have not been diagnosed with NAFLD yet, it is crucial to determine how to prevent it. Our study found a significant reduction in NAFLD risk if obesity was improved. This supports their findings in another direction, which offers an effective prevention method. In addition, there was a linear dose–response relationship between the duration of improvement in MHO and MHNO and the reduced risk of NAFLD. The duration of improvement was grouped into four categories: 0 months, 1–12 months, 13–24 months, and over 25 months. NAFLD risk did not differ significantly between the MHO–MHNO consistent improvement group and the intermittent improvement group. This may be due to the small sample size of the group with consistent improvement. Indeed, previous studies have assessed the effect of weight loss on patients with NAFLD, and the guidelines recommend at least 3%–5% weight loss to improve hepatic steatosis in patients with NAFLD (6). In Cho’s study, they first demonstrated that even weight loss of 1% may help reduce the risk of developing hepatic steatosis in MHO individuals without NAFLD (19). Our study suggests that obesity has a greater impact on NAFLD than metabolic abnormalities, supporting the importance of weight loss when an individual is obese without the presence of NAFLD. Unfortunately, our previous data indicate that obesity among the health check-up population has not decreased (20). Considering the fact that weight control is particularly prominent among Chinese, our findings have significant clinical implications.

As predicted, MUNO individuals are at a greater risk of NAFLD than MHNO individuals. In our study, approximately 40% of MUNO individuals improved to MHNO status, reducing the risk of NAFLD even with intermittent improvements. Furthermore, linear dose–response relationships were identified between improved health status from MUNO to MHNO and a reduced risk of NAFLD. It is noteworthy that men, older individuals, and those requiring MetS medication were observed to have a lower NAFLD risk among individuals who switched from MUNO to MHNO. Previous studies demonstrated that multiple molecular pathways and gene networks implicated in lipid metabolism, insulin signaling, and inflammation show sexual dimorphism (28, 29). Meanwhile, aging was generally correlated with increases in insulin resistance and the prevalence of the metabolic syndrome (30). In light of this difference, it may be explained why male subjects and older individuals may benefit more from the improvement of metabolic abnormalities.

We found that MUO is more susceptible to NAFLD than MHO. Compared to MHO, MUO has more abdominal and visceral fat accumulation, less physical activity, more sedentary activities, and poorer CRF, which will lead to an increased risk of NAFLD (23, 31–37). Therefore, when intervening in the MUO population, we should not only focus on weight loss but also improve fat distribution and promote cardiopulmonary function. A reduction in the risk of NAFLD can be achieved to a greater extent by improving both metabolic status and obesity. A linear dose–response relationship was found between the duration of improvement from MUO to MHNO and the reduction in NAFLD risk.

Our study has several strengths, including the large population-based cohort, the longitudinal design, and follow-up over 148,186 person-years, allowing the assessment of NAFLD risk according to improvement in metabolic health and/or loss of weight in Chinese subjects without NAFLD at baseline. As far as we know, we were the first to report that improving metabolic abnormalities and/or losing weight in different subgroups have been beneficial. It is expected that these findings will have significant clinical implications for the prevention of NAFLD in the Chinese population. Moreover, our findings could pave the way for further research into the most effective ways to prevent NAFLD in other populations. It is our hope that our research will contribute to a better understanding of the underlying causes of NAFLD and ultimately lead to more effective treatments and preventive measures for this potentially life-threatening condition. Furthermore, we had extensive data on health behavior and sociodemographic information, allowing us to examine the relationship between metabolic health and/or body weight and NAFLD under the adjustment of a wide range of confounders.

Results should also be interpreted with consideration for limitations. Despite the large sample size, our study was not a community-based study but rather a cohort study based on the health checkup population. Moreover, the subjects enrolled in the present study are predominantly from urban areas. Due to these limitations, our subjects were not generalizable to the general Chinese population. More cohorts of a mixture of urban and rural populations should be used to validate our findings. The results of this study may still be confounded by the fact that we did not collect all of the information that could have influenced outcomes, including information about healthcare and household income relating to NAFLD onset. In addition, insulin sensitivity is not routinely assessed during health check-ups, and C-reactive protein is not routinely measured. We did not use the combination of these two variables as a measure of metabolic health. This would make our measurement of changes in metabolic exposure less sensitive than when we used the metrics described above. However, we used exposure measurements in the same manner throughout the population, even if some misclassification was present, which would result in more conservative findings.

## Conclusion

This study proves that MHO, MUNO, and MUO had a higher NAFLD risk than MHNO. Importantly, we also found that no matter whether the improvement in metabolic status and/or loss of weight was intermittent or persistent, it could reduce NAFLD risk, independent of traditional risk factors.

## Data availability statement

The datasets presented in this article are not readily available because of ethical and privacy restrictions. Requests to access the datasets should be directed to the corresponding author.

## Ethics statement

The studies involving human participants were reviewed and approved by the Ethics Committee of the Third Xiangya Hospital. Written informed consent to participate in this study was provided by the patients/participants or patient/participants’ legal guardian/next of kin.

## Author contributions

XH: funding acquisition, methodology, and writing original draft preparation. WO: formal analysis, manuscript editing, and interpreting results. YHu, YHe, BT, PY, and KC: investigation. YHe: supervision. HW, QL, and XL: experimental work. LY: formal analysis. JD: interpreting results. YL: supervision, funding acquisition, methodology, writing, reviewing, and interpreting results. All authors contributed to the article and approved the submitted version.
