# Supplementary material for: The effects of weight loss and improved metabolic health status on the risk of non-alcoholic fatty liver disease—results from a prospective cohort in China
Source: Front Nutr. 2023 Nov 28;10:1239996. doi: 10.3389/fnut.2023.1239996 (PMC10718644; doi:10.3389/fnut.2023.1239996)
Supplement: Supplementary file 1 [file Data_Sheet_1.docx]

**Supplemental Material**

**Supplemental file 1: Methods for clinical definition and measurements**

**Supplemental file 2: Figure S1 RCS logistic models for estimating risk of NAFLD and duration of improvement in weight and metabolic health during follow up**

**Supplemental file 1: Methods for clinical definition and measurements**

**Clinical characteristics**

Blood pressure (BP) was measured on the right upper arm in the sitting position after 10-15 min of rest using a validated digital automatic analyzer (Omron 9020). Systolic BP and diastolic BP were each measured twice and the mean of the two readings was considered in the analysis. If the two readings differed by >5mmHg, a third measurement was performed and the average of all three readings was applied.

Data on demographic variables, medical history, lifestyle, and social status (marriage, education, occupation, and annual income) were collected by standardized questionnaires in Hunan. The data collection in Beijing is done by the medical examiner, and the records include: medical history and lifestyle.

**Definition of chronic diseases**

Hypertension was defined as self-reported hypertension diagnosed by a physician, self-reported regular use of antihypertensive medications, or systolic/diastolic blood pressure at recruitment ≥ 140/90 mmHg.

Dyslipidemia was defined as meeting any of the following criteria: 1) TC≥6.22 mmol/L; 2) LDL-C≥4.14 mmol/L; 3) HDL-C<1.04 mmol/L; 4) TG ≥2.26 mmol/L; 5) self-reported dyslipidemia or use of lipid-lowering medications;

Diabetes mellitus was defined as self-reported diabetes diagnosed by a physician, self-reported regular use of antidiabetic medications, or fasting glucose at recruitment ≥ 7.0 mmol/L.

**Laboratory measurements**

Fasting venous blood samples were collected and immediately processed and analyzed at the clinical laboratory of Aerospace Center Hospital and Third Xiangya Hospital, respectively. The automated analyzer (Beckman AU5811; Beckman, America) and (Hitachi 7600-110; Hitachi, Japan) were used in Aerospace Center Hospital and Third Xiangya Hospital, respectively.

Fasting blood glucose was measured with the glucose oxidase method. Reagent test kit: DiaSys Diagnostic before July 2019, LEALEADMAN to date in Aerospace Center Hospital; Hitachi in Third Xiangya Hospital. HDL cholesterol and triglycerides were measured with enzymatic methods. Reagent test kit: DiaSys Diagnostic before July 2019, MC bioengineering to date in Aerospace Center Hospital; Hitachi in Third Xiangya Hospital. The fasting venous blood test data were calibrated when the reagent test kits were changed in Aerospace Center Hospital. All performance of sample analysis was in accordance with the manufacturer's specifications.


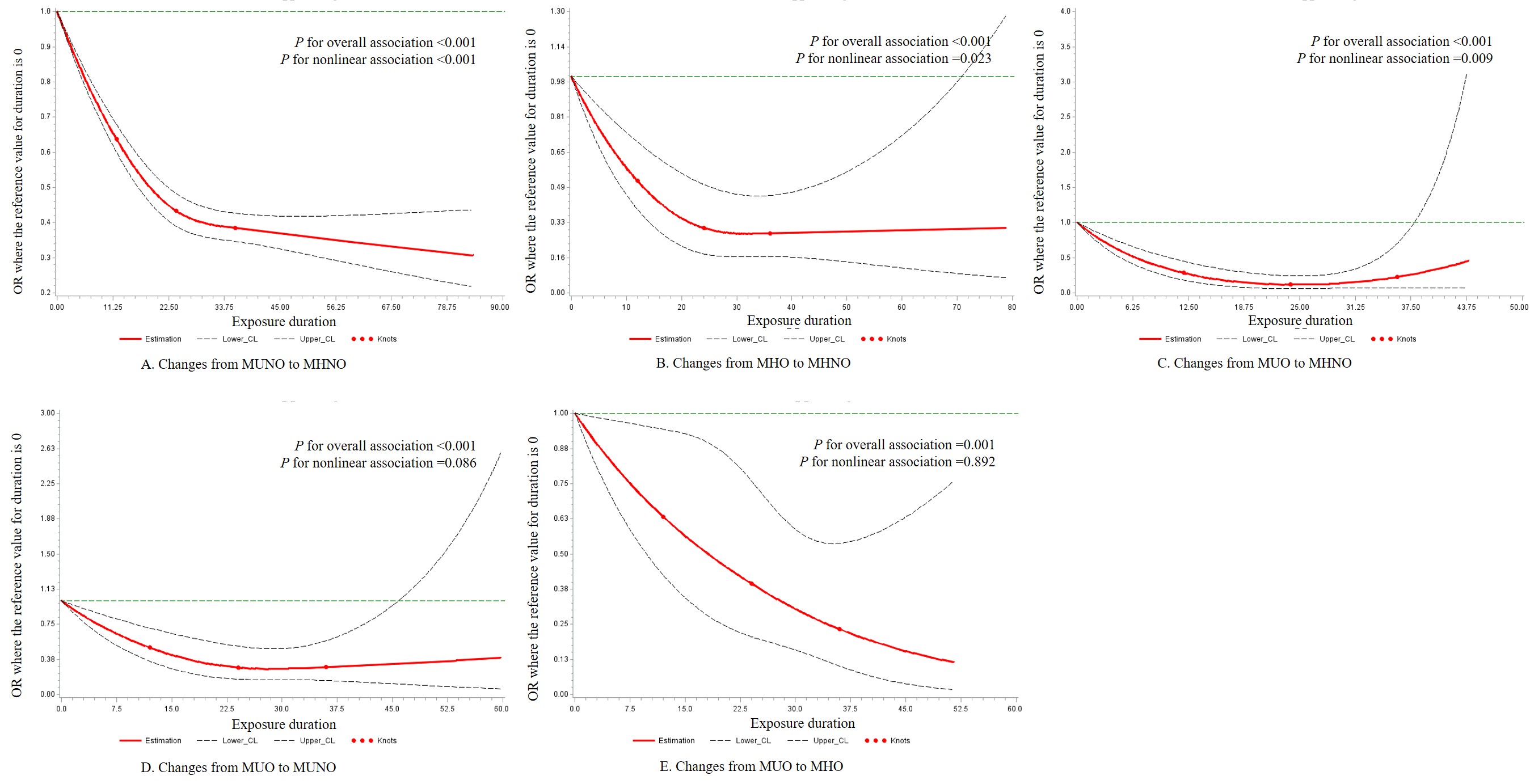


Figure S1. RCS logistic models for estimating risk of NAFLD and duration of improvement in weight and metabolic health during follow up

Exposure duration was coded using an RCS function with three knots located at the 12, 24 and 36 months. *Y*-axis represents the odds ratio for risk of NAFLD with duration at zero level to serve as a reference.. Referenced group in (A) was MUNO throughout, and in (B) was MHO throughout, and in (C)- (E) was MUO throughout. Dashed lines are 95 per cent confidence intervals. Knots are represented by dots. All the estimations were based on adjustment for sex, age, alcohol usage, smoking, location of enrollment location, and LDL cholesterol.

Abbreviation: NAFLD, non-alcoholic fatty liver disease; MHNO, metabolic healthy non-obese; MUNO, metabolic unhealthy non-obese; MHO, metabolic healthy obese; MUO: metabolic unhealthy obese; OR, odds ratio; CI, confidence interval.
